# A Bipartite Obturator Artery with Multiple Pelvic Branching—A Gynecologic Approach

**DOI:** 10.3390/diagnostics12112614

**Published:** 2022-10-27

**Authors:** Jailenne I. Quiñones-Rodríguez, Alexandra N. Acevedo-Arroyo, Camille L. Santiago-Negrón, Lucia F. Garcés-Torres, Carlos Fonseca-Salgado

**Affiliations:** 1 Department of Clinical Anatomy, Sam Houston State University College of Osteopathic Medicine, Conroe, TX 77304, USA; 2 Department of Anatomy and Cell Biology, Universidad Central del Caribe School of Medicine, Bayamón, PR 00960, USA; 3 Center for BioMedical Visualization, Department of Anatomical Sciences, St. George’s University School of Medicine, St. George FZ818, Grenada; 4 Obstetrics and Gynecology Department, San Juan City Hospital, San Juan, PR 00921, USA

**Keywords:** obturator artery, anatomical variations, accessory obturator artery

## Abstract

Background: The obturator artery (OA) often presents multiple anatomical variations. These can be an atypical origin, variable anastomosis, or abnormal course within the pelvis. Methods: This study aimed to report a rare arterial variation in a Puerto Rican female cadaver that showed two abnormal obturator arteries with multiple pelvic branches. The OA emerged from the anterior branch of the internal iliac artery, which typically runs anteroinferior along the lateral wall of the pelvis to the upper part of the obturator foramen. Results: The atypical OA described in this report provided two variant branches. Abnormal obturator artery I (AOAI) emerged first and gave rise to three additional branches, while abnormal obturator artery II (AOAII) emerged second and gave rise to two other branches. Conclusions: Identifying these accessory arteries is essential for surgical interventions, particularly within the field of gynecology and urogynecology. Knowledge regarding anatomical variations within this region must be assessed preoperatively to decrease the risk of iatrogenic injury.

## 1. Introduction

The vascular distribution of the obturator artery (OA) is crucial during surgical interventions within the pelvis, such as those involving femoral hernias [1]. The latter is also of utmost importance during gynecologic, trauma, or orthopedic procedures near the lateral wall of the lesser pelvis [2,3]. Injuries to the OA may present in cases of trauma involving pelvic fractures, which are the most common cause of hemorrhage within the pelvis [4]. Injuries to the OA may also present in cases of pelvic gunshot wounds, although rare.

The OA is a parietal pelvic branch of the anterior division that runs anterior-inferiorly on the lateral pelvic wall [5]. Before entering the obturator foramen accompanied by the obturator nerve (ON) and obturator vein (OV), the OA gives rise to pubic, vesical, and iliac rami [6]. The pubic ramus of the OA anastomoses with a pubic ramus from the inferior epigastric artery (IEA), the vesical ramus supplies the medial urinary bladder, and the iliac ramus supplies the ileum and iliacus muscle. Following its course through the obturator foramen, the OA continues into the medial compartment of the thigh, where it divides into anterior and posterior branches, subsequently exiting the pelvis.

Variations regarding the OA are of clinical importance and of consideration for gynecological and urological procedures. Unawareness of such variation could lead to iatrogenic injuries during surgical procedures. For instance, one of the most important causes to consider is major obstetric hemorrhages as one of the main causes of maternal mortality [7]. Major hemorrhages can occur in pregnant women during antepartum, delivery, or in the postpartum period. Anatomical variants of the OA could be a potential source of maternal hemorrhage in these women. In such cases, maternal hemorrhage may be controlled by pelvic vessel ligations (internal iliac, uterine, hypogastric, or ovarian arteries), and/or embolization [8].

Embryologically, variations of the OA have been hypothesized to be due to an unusual selection of channels from a primary capillary plexus that establishes the final arterial pattern [9,10]. Awareness of such an aberrant blood supply is essential, as these may involve unexpected and undesired outcomes during surgical procedures. In this case report, we describe a variation in a Puerto Rican female cadaver that showed two abnormal OAs with multiple pelvic branches.

## 2. Case Report

**Figure 1:**
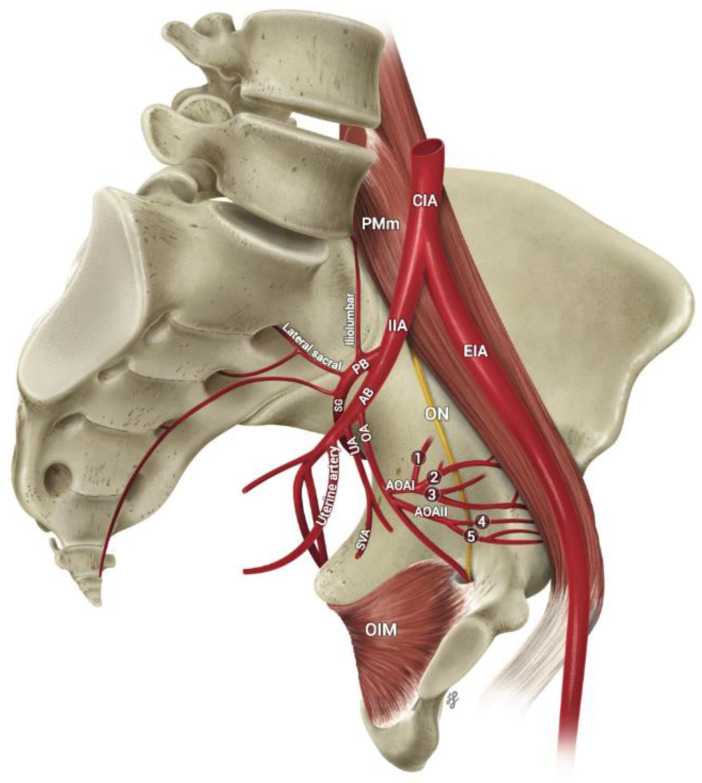
Illustration showing the atypical findings in the pelvic region presented in the case report. The common iliac artery (CIA) passes through the psoas major muscle (PMm) and divides into the external iliac artery (EIA) and internal iliac artery (IIA). The artery travels along the obturator fascia of the obturator internus muscle (OIM) of the pelvic sidewall, between the obturator nerve (ON) and obturator vein. A variation was found with multiple branches in its course before leaving the pelvic cavity through the obturator foramen. The anterior branch (AB) of the internal iliac artery (IIA) provided the obturator artery (OA) branch, divided into two atypical OA-provided anatomically variant branches. Abnormal obturator artery I (AOAI) gave rise to three branches (1, 2, 3), while abnormal obturator artery II (AOAII) gave rise to two branches (4, 5). In addition, anterior branches (AB) such as the umbilical artery (UA), superior vesical artery (SVA), uterine artery and other branches from the posterior branch (PB), superior gluteal artery (SG), lateral sacral artery, and iliolumbar artery were anatomically oriented.

During a gross cadaveric dissection session, an anatomical variation was found in an elderly adult female cadaver with proliferous pelvic branching prior to exiting the pelvic cavity through the obturator foramen (Figure 1). The clinical history, family history, and cause of death were unavailable.

**Figure 2:**
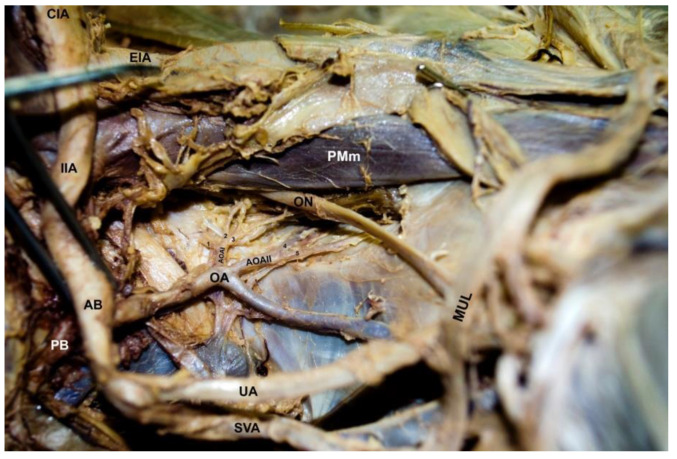
Variation of branching found in the pelvic region. The common iliac artery (CIA) divides into the external iliac artery (EIA) and internal iliac artery (IIA). The IIA divides into the anterior branch (AB) and posterior branch (PB). The AB gives rise to the obturator artery (OA), which provides the atypical obturator arteries (AOAI and AOAII). The AOAI gives rise to AOAI—1, 2, 3 and AOAII—4, 5. In addition, we observe the obturator nerve (ON), umbilical artery (UA), medial umbilical ligament (MUL), superior vesical artery (SVA), and psoas major muscle (PM).

Female pelvic cavity dissection was performed following Grant’s Dissector. After complete visualization of the pelvic viscera and adnexa, they were removed. The pelvis was sectioned in the midline to proceed with the study of the hemipelves. Upon visualization of the common iliac artery (CIA), pelvic vascular components were evaluated. Examination of the right hemipelvis showed an external (EIA) and internal iliac artery (IIA) following their respective courses. After coursing over the pelvic brim, the IIA was divided into an anterior and posterior division (Figure 2), with the anterior branch giving off the obturator artery (OA) followed by the umbilical artery (UA) obliterated to the median umbilical ligament (MUL), superior vesical artery (SVA), middle rectal artery (MRA) *, inferior gluteal artery (IGA) *, internal pudendal artery (IPA) *, visceral vaginal artery (VA) * and uterine artery (UtA) * (* not visualized in figures). Halfway through its course, in the lateral pelvic wall, the OA gave off two abnormal lateral branches, AOAI and AOAII (Figure 2). First, AOAI was subdivided into three smaller branches, 1–3, and then AOAII was subdivided into two smaller branches, 4 and 5 (Figure 2). The AOA proceeded on its course, piercing through the obturator fascia, where it was most likely to anastomose and contribute to the femoral head blood supply. Further investigation will evaluate the anastomosis of this AOA. The obturator nerve (ON) and obturator vein (OV) were anatomically normal, accompanied by the OA to exit through the obturator foramen. No vascular anomalies were observed on the left OA or other IIA system vessels.

## 3. Discussion

The OA has been well documented to present significant anatomical variability. The atypical origins of OA variations have been extensively described and correlated to hemorrhagic risks in surgeries involving the pelvis [11,12,13,14,15,16,17,18,19,20,21,22,23]. According to a meta-analysis by Sañudo et al., the origin of OA variations could arise from the anterior or posterior trunk of the IIA, the inferior epigastric artery (IEA), the external epigastric artery (EEA), or the femoral artery [24]. Previous reports have established that the incidence of these variations in their origin ranges between 6.6–63.63% [14]. Moreover, the anastomosis between the OA and the EIA or the IEA, known as the “corona mortis”, has mainly been reported due to the potential complications during general, orthopedic, or gynecologic surgeries [25,26,27]. The incidence of the “corona mortis” ranges from 10–43% [27].

### 3.1. Embryological Basis

The embryological basis for abnormalities in the arterial patterns of IIA vessels is theorized to be the result of an unusual selection of channels from a primary capillary plexus [8,9,10]. The final arterial pattern, normal or variant, is determined by the primitive vessels, which may enlarge or retract and disappear [9]. Others have proposed that the underlying process for branching pattern variations may originate from hypoxia inducing the expression of angiogenic factors, such as vascular endothelial growth factor (VEGF) and nitric oxide synthase, which stabilize protein complexes that lead to the abnormal vessel formation [10].

While the embryological basis for OA anatomical variations of origin and anastomoses can explain the patterns observed, we propose that AOAI and AOAII could be the result of the development of collateral branches to fulfill the demand of pelvic structures, insufficiently supplied by other vessels. These inadequate IIA system vessels may have underlying vascular pathologies associated with aging.

### 3.2. Clinical Significance

Anatomical variations in the pelvic blood supply are common [11,12,13,14,15,16,17,18,19,20]. Thus, a detailed description of these variants has been an advancement promoting minimally invasive gynecologic surgery and surgical quality and safety. Gynecologic surgery is at the forefront, with the introduction of laparoscopy as a minimally invasive approach for adnexal surgery. One of the potential spaces in close proximity to OA variants is the vesicovaginal space, which is exposed for interventions such as vesicovaginal, uterovaginal fistula repair, urinary incontinence procedures, transvaginal cystocele, and laparoscopic hysterocolpopexy [28].

The dissection of the vesicovaginal space exposes the cranial and caudal portion of the vesico-uterine ligament, allowing the exposure of vessels and nerves, described by Fujii [29]. Based on the proximity of the OA branches during vaginal, laparoscopic, and robotic hysterectomy, it was reported that variant branches of the OA could be at risk of injury during urogynecology procedures to manage stress incontinence (SUI) [12]. SUI is a common pelvic floor disorder, with an incidence from 15 to 80% of women worldwide [30]. Most patients with moderate to severe SUI need surgical treatment [31]. The Burch colposuspension is a surgical technique used to manage stress urinary incontinence using laparotomy or laparoscopy [32]. During this procedure, the surgeon places two bilateral sutures that fix the urethra to Cooper’s ligament. Although the “gold standard” is a minimally invasive mid-urethral sling, the Burch procedure remains an option for stress urinary incontinence.

Anatomically variant branches can also be at risk during other urogynecological procedures for vaginal wall descent, uterovaginal prolapse, and neovaginal reconstruction [12]. Tension-free vaginal tapes (TVT) are widely used to treat stress urinary incontinence [33]. Currently, there is a renewed interest in the use of sling techniques, as a greater understanding of their mechanism has developed and means of avoiding some of the pitfalls have been discovered. The classical sling procedure involves creating a hammock effect on the bladder’s neck [34,35]. The advantages of this procedure are to further reduce the invasiveness of the surgical procedures by avoiding the passage of the needle carriers through the retropubic or obturator regions, minimizing the risk of iatrogenic injury [36]. The correct placement of the TVTs with a new obturator approach offers additional safety during the transobturator course, being directed away from the urethra and bladder and unable to expose the anterior branch of the obturator artery, when present.

These variants could be also at risk during gynecologic oncology procedures, such as lymphadenectomy in the obturator and external iliac region. Identification of obturator artery vascular variations in the pelvic region has multiple surgical applications in gynecology and should be considered during the preoperative course. Proper identification of these vascular abnormalities can decrease the risk of intraoperative complications such as hemorrhage. Bilateral internal iliac artery (IIA) ligation is a life-saving procedure which significantly reduces the pulse pressure and rate of blow flow caused by a hemorrhage, subsequently allowing effective thrombosis within small bleeding vessels, such as the OA [37]. After ligation, the pubic branches of the obturator artery anastomose with the inferior epigastric artery, a branch of the external iliac artery [38]. Some different indications regarding the IIA ligation are uterine laceration during endovascular repair of aortoiliac arterial aneurysm, radical hysterectomy or exenteration, postpartum hemorrhage secondary to abruption or placenta previa, and—as stated earlier—profuse pelvic hemorrhage from fracture of the pelvis or gunshot injury to the pelvis [37]. Pelvic arterial embolization is an effective treatment for intractable pelvic hemorrhages, including the IIA branching pattern where arteriographic facilities are available [39].

Pelvic surgeons should be aware of the anatomical variations regarding the IIA branching pattern while performing procedures such as the ones discussed in the reported study. This will potentially reduce the risk of iatrogenic injuries and subsequent long-term complications. Tracing along the neurovascular bundle (obturator artery, vein, and nerve) within the obturator foramen is an anatomic landmark that indicates an adequate inferior dissection and the course of the neurovascular bundle in the preperitoneal space.

Among the limitations of the presented case was the unavailability of clinical and family history, as well as the cause of death.

## 4. Conclusions

We report for the first time an abnormal OA with proliferous pelvic branching before exiting the pelvic cavity through the obturator foramen. Halfway through its course, within the lateral pelvic wall, two abnormal branches were identified, AOAI and AOAII. AOAI was further subdivided into three small branches, while AOAII was subdivided into two small branches. Based on the anatomical position of these lateral branches, we suggest they might be anastomosing with external iliac arteries, thereby providing collateral circulation.

Currently, no extensive reports have focused on the obturator vasculature variants in the elderly female population. Advancing age and predisposing vascular pathologies may be responsible for the development of collateral vessels, such as the ones previously reported, contributing to the OA findings in this population.

Furthermore, clinical anatomy studies of pelvic vasculature among this population are strongly encouraged to advance pubic surgical procedures, such as internal fixation of pubic fracture, an inguinal hernia repair, or other gynecologic interventions discussed in the reported study, in order to prevent adverse surgical outcomes among patients.

## Data Availability

Not applicable.
